# New Insights into the Application of High-Pressure Processing and Storage Time and Temperature to Sliced Iberian Chorizo

**DOI:** 10.3390/foods12030472

**Published:** 2023-01-19

**Authors:** Ana I. Carrapiso, Antonia Trejo-Álvarez, María Jesús Martín-Mateos, Jonathan Delgado-Adámez, Jesús García-Parra, Rosario Ramírez

**Affiliations:** 1 Tecnología de Alimentos, Escuela de Ingenierías Agrarias, Universidad de Extremadura, Ctra de Cáceres s/n, 06007 Badajoz, Spain; 2 Technological Agri-Food Institute (INTAEX), Centro de Investigaciones Científicas y Tecnológicas de Extremadura (CICYTEX), 06071 Badajoz, Spain

**Keywords:** sliced Iberian chorizo, high pressure processing, storage temperature, water activity, pH

## Abstract

Producing dry-cured meats with relatively high *a_w_* and pH allows companies to cut costs to the detriment of microbial control. The purpose of this study was to evaluate for the first time the effect of High Processing Pressure (HPP) and storage temperature on the microbial counts, instrumental color, oxidation and sensory characteristics of sliced Iberian chorizo with high *a_w_* and pH. First, 600 MPa was applied for 480 s to sliced chorizo with *a_w_*: 0.88 and pH: 6.01, and the treated and untreated samples were stored at 4 or 20 °C for 90 or 180 days. HPP, storage time and storage at 20 °C were successful at decreasing the microbial counts that were initially high. HPP and the storage temperature had a limited detrimental effect, whereas the storage time had a marked adverse effect on oxidation and some sensory traits. Despite the high *a_w_* and pH, no safety issues arose initially or during the storage at 4 or 20 °C. In conclusion, for chorizo with high *a_w_* and pH favoring high microbial counts, HPP may be an effective hurdle without a noticeable detrimental effect, and the economically convenient storage at 20 °C might be beneficial despite causing moderate quality loss.

## 1. Introduction

Iberian chorizo is a greatly appreciated dry-cured sausage made of minced Iberian pork (a rustic breed in the Iberian Peninsula) and spices, providing a specific flavor, natural antioxidants and antimicrobial components [1]. During chorizo ripening, the total microbial counts increase rapidly, both in chorizo with and without starter cultures, up to counts of 10^9^ CFU g^−1^ for the mesophilic aerobic bacteria and 10^8^–10^9^ CFU g^−1^ for the lactic acid bacteria (LAB) [2,3]. Similar counts are reached during ripening in other dry-cured sausages, for example 10^8^–10^9^ CFU g^−1^ for the LAB and around 10^7^ CFU g^−1^ for staphylococci, which prevail over other microorganisms such as molds (10^5^–10^7^ CFU g^−1^ in some sausages varieties) and yeasts (10^3^–10^5^ CFU g^−1^) [4]. Iberian chorizo is generally ripened until water activity (*a_w_*) and pH reach 0.70–0.80 and 5.3–5.8, respectively [5,6,7], through dehydration and fermentation. This results in the microbial stabilization of the sausages and in a decrease in the total counts depending on the sausage variety and manufacturer settings, with a decrease in LAB counts up to the 10^4^–10^8^ CFU g^−1^ range in spontaneously fermented sausages [8].

Some companies set higher values for the final *a_w_* and pH to reduce the maturation time, which produces economic benefits. A higher *a_w_* allows companies to cut costs through smaller water losses, and a higher pH better fits consumer demand for low acidity in Mediterranean dry-cured sausages [9], especially in high-quality traditional products. These products have fewer effective hurdles against microbial survival and growth, and are more likely to maintain the high counts reached during ripening. Although this may introduce new spoilage and food safety concerns to deal with, little information is available about Iberian chorizo with relatively high *a_w_* and pH.

High-Pressure Processing (HPP) has been proposed to minimize safety risks in chorizo [6,10,11] and other dry-cured products [12] due to its suitability to inactivate *Listeria* spp. without a drastic quality loss. However, most studies on Iberian chorizo have been performed on products with *a_w_* and pH within the usual ranges. The only study available on high *a_w_* and pH Iberian chorizo was not performed on sliced chorizo but on whole and half pieces, and showed that HPP increased the initial counts of mesophilic aerobic bacteria and lactic acid bacteria, challenging the feasibility of HPP to reduce the total microbial counts [13]. The increasing importance of sliced chorizo and the additional steps involved (casing removal, slicing and packaging) that favor quality loss and contamination with ubiquitous pathogens make it necessary to research the convenience of HPP to control the microbial counts in sliced chorizo with high *a_w_* and pH.

Sliced dry-cured meat products are normally stored at refrigeration temperatures to preserve quality and extend the shelf-life, since higher temperatures might favor quality loss, increasing oxidation [5]. However, retail stores frequently display and sell them at room temperature for marketing convenience, which might cause safety concerns involving some microorganisms. However, recent research has shown that storage at room temperature causes a faster drop in *Listeria* spp. counts than when under refrigeration [6,14]. Despite the potential safety concerns, little information on the effect of storage temperature on chorizo with high *a_w_* and pH is available [13], and there is no information for those chorizos as sliced products.

HPP may be an effective obstacle against microorganisms without having a noticeable detrimental effect on the quality; however, the physical-chemical characteristics of fermented products may alter the effect. In addition, the economically convenient storage at 20 °C might be beneficial from an economical point of view despite causing moderate quality loss. The study was based on the hypothesis that HPP and storage at 20 °C may provide an effective obstacle against the microorganisms without having a considerable detrimental effect on the product. Therefore, the main purpose of this study was to evaluate for the first time the effect of HPP and storage temperature on the microbial counts, instrumental color, oxidation and sensory characteristics of sliced Iberian chorizo with relatively high *a_w_* and pH.

## 2. Materials and Methods

### 2.1. Samples

Vacuum-packaged sliced Iberian chorizo was purchased from a leading company just after packaging. Following its usual procedure, it was made of a lean (70%) and fat (30%) pork mixture from Iberian pigs fattened outdoors and indoors (usual practice to produce Iberian chorizo), salt, spices (red dried paprika, garlic), other ingredients (sugar, lactose, milk powder, dextrin, dextrose), and additives (sodium nitrite, potassium nitrate, sodium citrate and diphosphates). The batter was kept under refrigeration for one day, stuffed into artificial casing with a caliber of 62–65 mm, and matured initially at 5–7 °C and 70–80% RH, and then by natural drying for a total of 90 days. The chorizos were sliced (2–3 mm thickness) and vacuum packaged (100 g per package) using a Zermat Orved VM-18 equipment at -0.8 bar in 20 cm × 30 cm packages made of high-barrier polyester and polypropylene (20/70), with a 107 ± 10% µm thickness and an oxygen permeability of <10 cm^3^ m^−2^/24 h atm (23 °C, 50% RH).

### 2.2. Experimental Design

The packages were randomly allotted to the Control and the HPP (subjected to a high-pressure treatment) groups. After that, the packages were stored at two different temperatures (4 and 20 °C), with three sampling periods (initial, 90 and 180 days). Each analysis was performed on five packages from each batch, except for the initial analyses (physical-chemical parameters, proximate composition and fatty acid profile), performed only on the Control batch, which was not stored. In total, 100 packages of chorizo were used: 50 packages for the microbial and physicochemical analyses, and 50 packages for the sensory analysis.

### 2.3. High Pressure Processing Conditions

Samples were pressurized at 600 MPa for 480 s in a semi-industrial hydrostatic pressure unit with 55 L of capacity (Hiperbaric Wave 6000/55; Burgos, Spain). Initial water temperature inside the vessel was 10 °C. Time to reach 600 MPa was 230 s. The conditions were chosen to ensure the sufficient microbial inactivation of pathogens [15,16].

### 2.4. Initial Characterization (Water Activity, pH, Protein, Humidity and Fat Content)

The samples were minced. Then, water activity (*a_w_*) was measured using a Labmaster-*a_w_* meter (Novasina AG, Lachen, Switzerland). The moisture content was calculated by drying the samples in an oven at 104 °C until reaching constant weights. The pH was measured after homogenization with deionized water (1:10) using a Crison pH25+ pH-meter (Crison, Barcelona, Spain). Fat content was determined gravimetrically after extraction with chloroform:methanol (2:1) and protein content by using the Kjeldahl method. Five packages of the initial chorizos (Control group) were analyzed.

### 2.5. Fatty Acid Profile

The fatty acid methyl esters (FAMEs) were obtained after reaction with methanolic KOH and analyzed using a Hewlett Packard (model HP-5890A) gas chromatograph equipped with a flame ionization detector (FID), a 30 m FFAP-TPA fused-silica column (Hewlett Packard) with 0.53 mm ID and 1.0 μm film thickness. The injector and detector were maintained at 230 °C and the oven at 225 °C. The carrier gas was nitrogen at a flow rate of 15 mL min^−1^. The FAMEs were quantified using tridecanoic acid as internal standard. Response factors were determined for all fatty acids by injecting samples containing a known amount of FAME standard and tridecanoate. The FAME identification was based on the retention times of reference compounds (Sigma). Results were expressed as required by the current Spanish regulation [17].

### 2.6. Microbiological Analyses

Ten grams of each sample were taken aseptically and homogenized with 90 mL of peptone water (Merck, Darmstadt, Germany, 1.07043) in a laboratory blender (Stomacher^®^ 400 Circulator). Serial decimal dilutions were made in sterile peptone water, and 1 mL of appropriate dilutions were poured or spread onto total count and selective agar plates. Mesophilic aerobic bacterial counts, lactic acid bacteria (LAB), molds and yeasts, *Staphylococcus aureus*, *Clostridium perfringens*, *E. coli*, *Salmonella* spp. and *L. monocytogenes* analyses were performed according to the methodology previously described [18]. Chromocult agar (Merck) was used for coliforms, which were incubated at 37 °C for 24–48 h. Results were expressed as log_10_ CFU (colony forming units) g^−1^. The detection limit of the above techniques was 10 CFU g^−1^ for all the microbial groups except S. aureus (100 CFU g^−1^). The absence in 25 g was evaluated for *Salmonella* spp. and *L. monocytogenes*.

### 2.7. Instrumental Color

The instrumental color was determined using a Minolta CM-5 spectrophotometer with autocalibration (Minolta Camera, Osaka, Japan), using an illuminant/angle D65/10 ⁰, a measuring area of 30 mm, and the CIE Lab color space. The color coordinates L *, a * and b * were measured, and Chroma and the Hue angle were calculated as C * = (a *^2^ + b *^2^)^0.5^ and *h°* = tan^−1^ (b */a *), respectively. The measurements were performed on two slices randomly selected per package and the data were averaged.

### 2.8. Lipid and Protein Oxidation

Lipid oxidation was assessed by using the thiobarbituric acid reactive substances (TBA-RS) method [19], and results were expressed as mg malondialdehyde (MDA) kg^−1^ chorizo.

Protein oxidation was evaluated by measuring the carbonyl groups formed during incubation with 2,4-dinitrophenylhydrazine (DNPH) in 2N HCl [20]. Protein oxidation was expressed as nmol carbonyls mg^−1^ protein.

### 2.9. Sensory Analysis

Eight trained panelists took part in a quantitative-descriptive test with unstructured 10 cm scales and twelve descriptors: overall odor intensity, off-odor, saltiness, sourness, sweetness, spicy flavor, overall flavor intensity, cured flavor and rancidity (ranging from not perceptible to very intense), and lean color (from orange to dark red), hardness (during chewing, from very tender to very firm) and juiciness (during chewing, from not juicy to very juicy). All the sessions were performed in a test room with individual booths, according to the ISO 8589:2007 standard [21], at room temperature and with white fluorescent lighting, following the standard procedures for sensory analysis and with all the panelists informed of being involved in the evaluation of commercially available chorizo and consenting to the use of the resulting data. Two slices of chorizo from each package were presented on a plate to each panelist. About 100 mL of water at room temperature was also provided. Four packages were randomly assessed in each session, except for two sessions with three packages, with a total of 13 sessions.

### 2.10. Statistical Analysis

A three-way (high pressure processing, HPP, storage temperature and time) analysis of variance (ANOVA) with interaction was performed on the data from the stored samples to check the effect of the three-way interaction. For the parameters with significant interaction, an ANOVA Simple Main Effects test was performed. A two-way (high pressure processing, HPP, and storage temperature) analysis of variance (ANOVA) with interaction was performed on the data from the stored samples. Additionally, a one-way ANOVA was carried out to check the effect of HPP on the 0-day samples, and another one to check the effect of the storage time. The HSD Tukey’s test was applied to compare the mean values when the ANOVA or the ANOVA Simple Main Effects test showed a significant effect. A Principal Component Analysis was performed to check the overall effect of HPP and storage time and temperature. The analyses were performed by using the SPSS package, Version 21.0 (SPSS Inc., Chicago, IL, USA).

## 3. Results and Discussion

### 3.1. Initial Chorizo Characterization

The initial *a_w_*, moisture and pH (Table 1) were over the usual values, whereas the other parameters were within the usual range (Table 2, Table 3 and Table 4).

*a_w_* (Table 1) was over the values generally reported in Iberian chorizo (0.70–0.80), as it was the moisture content, usually below 25% [5,6,7]. Despite being high, *a_w_* was still below the 0.92 individual limit established in the European Commission Regulation 2073/2005 [22] to prevent *Listeria* spp. growth in ready-to-eat food. Conversely, pH was over the 4.4 individual limit, and also over the 5.0 limit when *a_w_* is not over 0.94. Therefore, the product was stabilized against microorganisms by *a_w_*, without a crucial contribution of pH. A relatively poor contribution of pH to the microbial stability is usual in the Mediterranean sausages [9] and Iberian chorizo [5,6], where pH is usually in the 5.3–5.8 range. In our study, the pH was over that usual range. The fat and protein content and the fatty acid profile were in line with results from previous studies [13].

With respect to chorizo safety, the initial counts for all the pathogens were under the detection levels (*S. aureus*, *Cl. perfringens*, *E. coli*) or absent in 25 g (*Salmonella* spp. and *L. monocytogenes*), and remained so in all the samples over time.

Regarding the other microorganisms, the initial counts (day 0, Control group) for the mesophilic aerobic bacteria and the lactic acid bacteria (LAB) (Table 2) were within the range reported by other authors for similar microbial groups in chorizo and other dry-cured sausages [8,9], being slightly under the values reported for Galician chorizo with and without starter cultures (10^9^ CFU g^−1^ for the mesophilic aerobic bacteria and 10^8^–10^9^ CFU g^−1^ for the lactic acid bacteria (LAB) [2,3]), but over the values usually reported in Iberian chorizo [5,6,13]. The initial counts in Table 2 (day 0, Control group) were high when compared to those usually reported in Iberian chorizo. The high counts might have been favored by the high *a_w_* and pH of the samples (Table 1) when compared to the usual values reported in Iberian chorizo. In this respect, it should be noted that the counts were comparable to those reported in other types of chorizo with comparable *a_w_* and/or pH values [2,8].

### 3.2. Effect of High Processing Pressure, Storage Temperature and Time

The one-way ANOVA performed on the day 0 samples (Control vs. HPP samples) showed a significant effect of HPP on the mesophilic aerobic bacteria and lactic acid bacteria (Table 2), and no effect on instrumental color, the oxidative parameters (Table 3) and sensory characteristics (Table 4).

Regarding the 90 and 180-day samples, the three-way ANOVA (HPP, storage temperature and time) revealed significant three and/or two-way interaction for all the microbial variables, which reveals that for them, the two-way (HPP x storage temperature) interaction differs between samples stored for 90 and 180 days (Table 5). For the other variables, no significant three-way interaction was found. For them, the three-way ANOVA showed no effect of the three factors on the instrumental color (interaction being significant only for Hue), a significant effect of HPP and time (90 vs. 180 days) on oxidation (with significant storage temperature x time interaction for the carbonyl content), and a marked effect of the storage time on the sensory traits, whereas the effect of HPP, storage temperature and interaction was slight (Table 5).

#### 3.2.1. Effect of HPP

The HPP was successful at decreasing the counts of the microbial groups which were abundant, namely mesophilic aerobic bacteria and LAB, but not the others (Table 2). The detrimental effect of HPP was limited, with no effect on the instrumental color (Table 3) and a slight effect on the lipid and protein oxidation parameters (Table 3 and Table 5) and the sensory characteristics (Table 4 and Table 5).

The mesophilic bacteria and the LAB counts were strongly affected by HPP initially and after 90-day storage at 4 °C (when the counts in the Control samples were in the 7.7–8.9 log CFU g^−1^ range), although on day 180 (when the counts in the Control samples were in the 4.9–6.7 log CFU g^−1^ range) only the LAB were affected, and only after storage at 4 °C, in all the cases HPP causing a drop in counts (Table 2). These results indicate that the marked initial effect of HPP is gradually lost over the storage time, which also causes a decrease in counts in both the Control and the HPP samples (Table 2). The results for the mesophilic bacteria count on day 180, without any remaining effect of HPP after the progressive decrease over time, suggest that at that moment the main factor limiting the counts in the Control and HPP samples is not the day 0 count but the environmental conditions, which are still suitable to allow 5.9–6.0 CFU g^−1^ in these samples with high *a_w_* and pH. This might imply that HPP, before or after storage, might not be sufficient to efficiently lower the count below 6.0 CFU g^−1^ in Iberian chorizo with those characteristics

The decrease in counts caused by HPP (especially effective when the counts were higher) indicates that the HPP can be useful to control those microorganisms in sliced Iberian chorizo with high *a_w_* and pH, especially initially and to a lesser extent over storage. Regarding the yeasts and molds and coliforms, the already low counts were not affected by HPP initially, nor were they over 90-day storage; the coliforms were not affected by HPP on day 180 either (Table 2). However, after 180 days at 20 °C, a significant effect of HPP appeared on the yeasts and molds, with counts under the detection limit.

The marked interaction among HPP, storage temperature and time shows the complexity of understanding the effect of the factors studied on the microbiological counts, since each microbiological group presents a different behavior, probably dependent on the optimal growth conditions of each group as well as on other intrinsic factors of the product such as the composition, the initial counts, etc.

A previous study on a similar but unsliced product reported an inconsistent effect of HPP on the mesophilic bacteria, the LAB and the molds and yeasts (no data available for coliforms) [13], in line with previous studies on Iberian chorizo with the usual *a_w_* and pH, HPP causing both a drop and a rise in the counts [5,6]. The differences among studies suggest that there may be underlying factors that modulate the effect HPP on the microorganisms. These factors might be related to the chorizo characteristics and the microbial susceptibility itself. Regarding the product characteristics, it has been suggested that they might be the cause of the differences in the effect of HPP between Iberian loin and sausage [23]. In this respect, the effectiveness of HPP is affected by food characteristics [12]. Microbial survival against HPP increases as *a_w_* drops [24], and also when pH increases from 4 to 6.6 [25], both in model systems. Therefore, the more efficient microbial inactivation found in this study on chorizo with high *a_w_* and pH than in previous studies on usual Iberian chorizo was expected. However, there is no clear evidence to suggest that pH and *a_w_* were decisive in HPP causing an increase or decrease in counts when applied to chorizo, since both trends have been found in chorizo with high *a_w_* and pH [13] and usual *a_w_* and pH [5]. Slicing is not likely to have a crucial influence on the effect of HPP, since both a significant HPP-induced decrease and increase have been reported in sliced [5,6] and unsliced chorizo [5,13].

With respect to microbial susceptibility to HPP, it differs according to the species, the growth phase and the inoculum level, with noticeable differences and even opposite trends [12]. In this respect, when HPP has been applied to chorizo with high initial mesophilic and LAB counts (generally over 6 log_10_ CFU g^−1^) and molds and yeasts (over 3.5 log_10_ CFU g^−1^), a consistent decrease has been found [5,13]. Conversely, data suggest that HPP can have no effect and even favor an increase in the microbial counts initially or over storage when counts are under those values [5,6,13]. In this regard, it is known that HPP activates some bacterial spores, a second HPP being recommended to inactivate the activated bacteria [26]. In addition, it has been suggested that HPP may activate mold ascospores [5], which may thrive when there is little microbial competition for resources and favorable environmental conditions. Microbial growth after the HPP might cause an increase in counts only noticeable when the counts are low, whereas when they are high the activation and/or growth may not be evident, especially when the lethal effect of HPP prevails over the HPP-induced activation effect. Therefore, for chorizo with high *a_w_* and pH favoring high microbial counts, HPP might result in a consistent decrease in microbial counts, as our results show.

The instrumental color was not affected by HPP at any sampling time (Table 3). These results match previous data from sliced Iberian chorizo with usual *a_w_* and pH values, both before and after storage [5,6]. Regarding chorizo with high *a_w_* and pH, both no effect on Portuguese chorizo and a significant effect on Iberian chorizo (only before storage on a*, b*, C* and *h°*) has been found in half pieces [13]. Therefore, the effect of HPP on chorizo, if any, fades away over storage. Those results suggest that chorizo color, regardless of slicing, *a_w_* and pH, might be remarkably stable against the harmful effect of HPP. This stability may be explained by the presence of paprika pigments, the main contributors to chorizo color instead of nitrosylmyoglobin [27].

The lipid and protein oxidation parameters were not affected by HPP just after the treatment (Table 3). However, after 90-day storage the effect of HPP on lipid oxidation was significant on the samples stored at 4 °C, although the effect was not strong enough as to be significant in the Tukey test (Table 3). The HPP-induced increase in MDA disappeared after additional storage. The slight effect of HPP is in line with previous studies on Iberian chorizo, which also reported no initial effect on slices and/or half pieces of Iberian chorizo, both with the usual [5,13] and high *a_w_* and pH [13]; after storage, both no effect on half pieces [5,13] and a slight effect on sliced chorizo (only significant on the carbonyl content on day 180 [5], and on the MDA and the carbonyl contents on day 120 [6]) have been reported. Results indicate that the effect of HPP on the oxidative status of sliced Iberian chorizo with high *a_w_* and pH is not marked, and suggest that a higher *a_w_* and pH is not a key parameter influencing the HPP-induced oxidative damage in this product.

The only sensory characteristic affected by HPP was hardness, only affected after 180-day storage at 4 °C (HPP caused an increase in it). None of the other sensory traits were affected by the application of HPP, neither just after the HPP or over storage (Table 4). This modest effect indicates that consumers might not notice any HPP-induced quality loss on sliced Iberian chorizo with high *a_w_* and pH. Previous studies on Iberian chorizo using a similar sensory test have shown a moderate effect of HPP on slices with the usual *a_w_* and pH [5] and also on half pieces with either usual and high *a_w_* and pH [5,13]. The higher stability in our study suggests that high *a_w_*, pH and slicing might either protect the slices against HPP-induced damage or result in a quality loss that masks the effect of HPP. Although further work is advisable to reject the hypothesis of greater stability in the sensory traits of chorizo slices with high *a_w_* and pH, and therefore its greater convenience for the Iberian chorizo industry to preserve the sensory quality, a protective effect of slicing is unlikely, since it causes discoloration and oxidation in Iberian products [28]. Therefore, it may be suggested that the effect of HPP might be unnoticed when high *a_w_* and pH products had suffered a slicing step prior to packaging.

#### 3.2.2. Effect of the Storage Time and Temperature

The storage time and temperature had a marked effect on the microorganisms (Table 2) and no effect on the instrumental color (Table 3). In addition, the storage time had a marked effect on oxidation (Table 3) and some sensory traits (Table 4), whereas the effect of the storage temperature on them was modest.

Regarding the microbial counts, both storage time and temperature had a significant effect when the counts were over 6 log CFU g^−1^ for the mesophilic bacteria and the LAB, and over the detection limits for the yeasts and molds and coliforms (Table 2). The mesophilic aerobic bacteria and the LAB were greatly affected by the storage time and temperature, with a decrease over time and lower counts after storage at 20 °C than at 4 °C (Table 2). The coliforms were also affected by the storage time (significant fluctuations in the 4 °C samples, and a consistent decrease to the detection level in the 20 °C ones) and by the storage temperature (only in the HPP samples after 180 days). The fluctuations might be due to changes in the environmental conditions, which include an initial drop in oxygen and marked changes in the microorganism counts over time (a decrease in the mesophilic bacteria and the LAB, and only for the HPP samples at 4 °C an increase in the yeast and molds). These variations in oxygen and microbial competition for resources might result in favorable conditions for the surviving coliforms, which might explain the significant increase from day 90 to day 180 in the 4 °C samples. Although more research on the effect of storage time at 4 °C is advisable, it can be inferred that the storage time does not result in a consistent drop in the coliform counts when the samples are stored at 4 °C.

The effect was weaker on the yeasts and molds (with initial counts close to the detection limit), in which the storage time did not cause any decrease but an increase in some samples instead (HPP 4 °C and control 20 °C groups). The storage temperature interacted significantly with the HPP after 90-day storage for the mesophilic bacteria, and after 180 days for the LAB and molds and yeasts.

Previous studies on unsliced Iberian chorizo with high *a_w_* and pH also reported a significant decrease in the mesophilic and LAB counts over storage, an increase in molds and yeasts (the initial counts were also under the detection level), and lower counts at 20 than at 4 °C [13] (Table 2). However, for unsliced Iberian chorizo with usual *a_w_* and pH (and low initial mesophilic, LAB and molds and yeasts counts), no decrease was found over time, but an increase instead, especially in the HPP-treated samples; in addition, although for the mesophilic bacteria the counts were lower after 90-day storage at 20 than at 4 °C, the trend was the opposite for the LAB after 180 days [5]. In chorizo slices with usual *a_w_* and pH no effect of storage time and temperature was generally reported on the mesophilic counts, although molds and yeasts generally decreased over time [5,6], with lower counts at 18 °C [6] or no effect of the storage temperature [5]. Previous studies in other dry-cured meat products have shown that storage time causes a decrease in microorganisms such as *Listeria* spp, especially at room temperature [14]. Differences among studies in the effect of storage time and temperature do not seem to be clearly related to slicing or *a_w_* and pH. Conversely, it should be noted that there was generally a decrease over storage when the initial counts were high, and also when the samples were stored at 20 °C. Therefore, as mentioned before for the effect of HPP, the initial counts have a marked influence on the effectiveness of storage time and temperature to reduce the microbial counts, with a less noticeable repercussion of slicing, *a_w_* and pH.

The results suggest that room temperature is efficient to control the microorganisms (including coliforms) in chorizo slices with high *a_w_* and pH, and that storage at both 4 and 20 °C results in a drop in counts when the initial counts are high. However, when counts are already low, storage time and temperature are not beneficial to reduce the counts and even a slight rise in some microorganisms (such as molds and yeasts and coliforms) can occur over time. In addition, it should be noted that in another sliced dry-cured meat product, namely Iberian loin, the effect of the storage temperature on the coliform counts was just the opposite even though the counts were relatively high (Carrapiso et al., submitted). This highlights the convenience of performing additional studies to unveil the factors that modulate the effect of storage temperature on coliforms to prevent potential safety issues.

The instrumental color was not affected by the storage time, nor was it affected by the storage temperature (Table 3). This is in line with the great color stability generally reported in previous studies on Iberian chorizo, with no effect or a slight effect of storage time regardless of *a_w_* [5,13], and a slight effect of the storage temperature on unsliced chorizo with high *a_w_* and pH [13], but a not fully consistent effect on the usual chorizo, ranging from no effect on slices and a slight effect on unsliced pieces [5] to a marked effect on thick slices, especially on the L* coordinate [6]. As mentioned above for microorganisms, differences among those studies are not clearly related to slicing, *a_w_* and pH, and may be attributed instead to additional factors, e.g., related to the company procedures during the chorizo production (e.g., paprika quantity or quality) and packaging parameters (e.g., O_2_ film permeability). It should be noted that previous work on other Iberian products has shown a marked effect of time and/or storage temperature on the instrumental color [23,29]. As mentioned before for HPP, the greater stability of chorizo during storage might be related to the presence of additional pigments from paprika (which is not added to products such as ham or salchichon) as the main contributors to color [27], masking the color of the storage-sensible meat pigments.

Regarding oxidation, the MDA and carbonyl content markedly increased over storage in all the groups, whereas the storage temperature hardly affected them (Table 3). The increase over storage was substantial, with most sample groups doubling or even trebling the initial values on day 180 (Table 3). This marked effect is in line with the results reported in high moisture content slices (*a_w_* not reported) [30] and half pieces with high *a_w_* and pH [13]. However, it differs from those from chorizo with usual *a_w_* and pH, where a slight increase in MDA and fluctuations in the carbonyl content were reported [5,6]. This suggests that high *a_w_* and pH might make the Iberian chorizo more susceptible to lipid and protein oxidation. Further studies are advisable to confirm this increased susceptibility.

Conversely, the effect of the storage temperature on oxidation was slight, with no effect on lipid oxidation and a significant effect of protein oxidation only on day 180 and in Control samples (Table 3). The results for lipid oxidation are in line with previous studies on sliced Iberian chorizo, which reported no effect [5] or a slight effect (only significant after 30 days but not after 60 and 120 days) [6]. This differs from studies on whole and half pieces, where a consistent effect on lipid oxidation was found after 180 days, regardless of *a_w_* and pH, with higher values after storage at 20 than at 4 °C [5,13]. Regarding the carbonyl content, the effect of the storage temperature was not significant after 90 days, but it was after 180 days in the Control samples, with higher values at 4 than at 20 °C (Table 3). This significant effect is not in line with previous results on sliced Iberian chorizo, which reported no effect or a lower content in the 4 °C- samples [5,6,13]. The higher carbonyl content might be explained by the consistently higher microbial counts in them. In addition, it should be noted that the values for lipid oxidation are in line with those reported in half pieces of similar Iberian chorizo (with the same category and from the same company [13]). However, protein oxidation was higher in the sliced product than in the half pieces (compare Table 3 and [13]). This indicates that slicing favors protein oxidation development, both initially and over storage in this type of chorizo.

**Figure 1:**
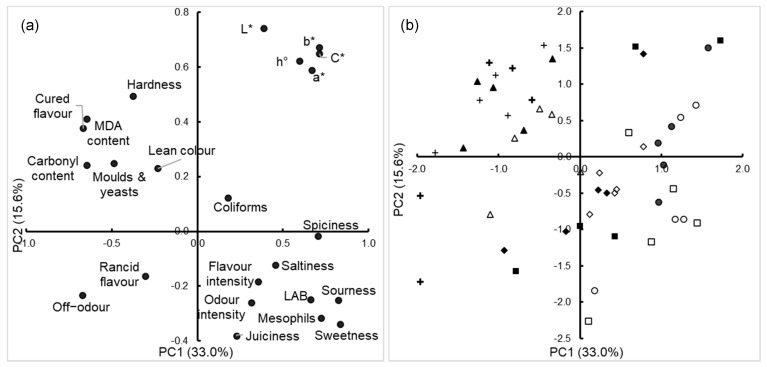
Projection of the variables (**a**) and samples (**b**) onto the space defined by the first two principal components (PC1/PC2) extracted from the variables in Table 2, Table 4 and Table 5. (**b**) ○: Control, day 1; ●: HPP, day 1; □: Control, 90 days at 4 °C; ■: HPP, 90 days at 4 °C; ◊: Control, 90 days at 20 °C; ♦: HPP, 90 days at 20 °C; Δ: Control, 180 days at 4 °C; ▲: HPP, 180 days at 4 °C; +: Control, 180 days at 20 °C; +: HPP, 180 days at 20 °C.

Regarding the sensory characteristics, the storage time resulted in marked changes, whereas the effect of the storage temperature was modest. The storage time affected mainly the taste and flavor traits, and only two traits (juiciness and rancidity) out of 12 were not affected by time in any sample group. This marked effect is in line with previous studies on Iberian chorizo [5,13]. The most affected traits were sourness, sweetness and spiciness (they decreased over time) and the cured flavor (increased over time) (Table 4). The changes in these traits might be related to a decrease in acids (mainly generated from the microorganisms, which decreased over time) and some compounds from the spices through chemical reactions that continued after the manufacture process. The variability in those sensory variables related to the storage time can be seen in Figure 1a, where they reached high scores (over 0.7 in all the cases) in the first principal component (PC1). It was the PC1 that showed a clear difference among the samples according to the storage time: the samples without storage reached high positive scores in the PC1, the 180-day samples reached high negative scores, and 90-day samples occupied an intermediate zone (Figure 1b). Figure 1a also shows that the global effect of the storage time was more marked than those of the storage temperature and HPP, which did not clearly influence the mapping of the samples on the space defined by the two first principal components.

The effect of storage temperature was only significant after 180 days and only on the lean color and hardness (lower scores at 4 than at 20 °C), juiciness (higher scores at 4 than at 20 °C) and flavor intensity (higher scores at 4 than at 20 °C, only in the Control samples) (Table 4), which might be due to the development of chemical reactions typical of the ripening stage. The slight effect is in line with results from previous studies on sliced and unsliced chorizo, regardless of *a_w_* and pH [5,13].

It should be noted that the effect of storage time and temperature on some characteristics (the decrease in sourness over time, the increase in the cured flavor over time and in the lean color and hardness at 20 °C) did not clearly cause a loss in the sensory quality but instead can be considered an improvement. Therefore, for Iberian chorizo with high *a_w_* and pH, the storage step might provide conditions to improve those traits and even complete the ripening process, restricting the typical water losses. Further research is needed to confirm if an additional final storage step during the manufacturing process for vacuum-packaged Iberian chorizo with high *a_w_* and pH might be convenient for the industry.

## 4. Conclusions

The application of HPP to sliced Iberian chorizo with high *a_w_* and pH caused a drop in the microbial counts, especially when they were high, and the effect persisted over storage. Conversely, the effect on color, oxidation and sensory traits was slight. Therefore, for chorizo with high *a_w_* and pH favoring high microbial counts, HPP might provide an effective and consistent decrease in the microbial counts without producing a noticeable detrimental effect, suggesting that no quality loss might be perceived by most consumers. Further research is advisable to rule out the possibility of other factors (such as spice quantity and quality, additives, etc.) cancelling out or even overturning the effect of HPP.

In addition, the relatively high *a_w_* and pH did not result in any safety issues initially or during storage and did not hinder a marked decrease in the microbial counts over time, especially at the higher temperature, although with some quality loss. Therefore, the economically convenient storage at room temperature might be beneficial.

## Figures and Tables

**Table 1:** Results for the physical-chemical parameters, proximate composition and fatty acid profile (mean ± standard deviation) of the sliced Iberian chorizo.

General Parameters	
*a_w_*	0.88 ± 0.01
Moisture (%)	31.48 ± 2.36
pH	6.01 ± 0.27
Fat (%)	27.63 ± 2.04
Protein (%)	25.13 ± 2.26
**Fatty acid profile (%) ^1^**	
C12:0	0.1 ± 0.0
C14:0	1.4 ± 0.0
C16:0	25.8 ± 0.1
C16:1	3.0 ± 0.1
C17:0	0.3 ± 0.0
C17:1	0.2 ± 0.0
C18:0	14.2 ± 0.3
C18:1	46.6 ± 0.3
C18:2	6.8 ± 0.4
C18:3	0.5 ± 0.1
C20:0	0.2 ± 0.0
C20:1	1.0 ± 0.0

^1^ Only those required by the current regulation [13].

**Table 2:** Microbial counts (log CFU g^−1^) (mean ± standard deviation) over the detection limit in sliced Iberian chorizo HPP (HPP) or not (Control), not stored (0) or stored at 4 or 20 °C for 90 or 180 days, and significance from a one-way (storage time) ANOVA, a one-way (T, HPP) ANOVA on day 0 data, and Simple Main Effects tests (day 90 and 180) (T, HPP; ST, storage temperature) ^1^.

	Storage (Days)	4 °C		20 °C		*p*-Value		
		Control	HPP	Control	HPP	T	ST	TXST
**Mesophilic aerobic bacteria**	0	8.9 a ^1^ ± 0.0	7.4 a ^2^ ± 0.5	8.9 a ^1^ ±0.0	7.4 a ^2^ ± 0.5	***	-	-
	90	8.3 b ^1^ ± 0.1	6.6 b ^2^ ± 0.2	6.6 b ^2^ ± 0.4	6.3 b ^2^ ± 0.2	***^,i^	***^,ii^	***
	180	6.0 c ± 0.1	5.9 c ± 0.1	6.0 c ± 0.1	6.0 b ± 0.1	ns	ns	ns
*p*-storage time		***	***	***	***			
**Lactic acid bacteria**	0	8.1 a ^1^ ± 0.1	7.5 a ^2^ ± 0.3	8.1 a ^1^ ± 0.1	7.5 a^2^ ± 0.3	***	-	-
	90	7.7 a^1^ ± 0.4	6.5 b ^2^ ± 0.4	5.7 b ^3^ ± 0.5	4.3 b ^4^ ± 0.4	***	***	ns
	180	6.7 b ^1^ ± 0.2	4.6 c^2^ ± 0.4	4.9 b ^2^ ± 0.6	4.2 b^2^ ± 0.6	***^,i^	***^,iii^	**
*p*-storage time		***	***	***	***			
**Yeasts and molds**	0	1.2 ± 1.1	<1 b	1.2 ab ± 1.1	<1	ns	-	-
	90	<1	<1 b	<1 b	<1	-	-	-
	180	2.2 ^1,2^ ± 1.3	3.1 a^1^ ± 1.7	2.1 a ^1,2^ ± 1.3	<1 ^2^	*^,iv^	*^,v^	**
*p*-storage time		ns	***	*	*ns*			
**Coliforms**	0	2.5 a ± 0.3	3.0 a ± 0.3	2.5 a ± 0.3	3.0 a ± 0.3	ns	-	-
	90	<1 b	<1 b	<1 b	<1 b	-	-	-
	180	1.4 a ± 1.0	2.7 a ^1^ ± 1.0	<1 b	<1 b ^2^	ns	**^v^	ns
*p*-storage time		**	***	***	***			

^1^ ns: *p* ≥ 0.05; *: *p* < 0.05; **: *p* < 0.01; ***: *p* < 0.001. a,b,c: different letters in the same column indicate significant differences over storage in the Tukey test. ^1,2,3,4^: different superscript numbers in the same row indicate significant differences between groups in the Tukey test. ^i^: *** only for 4 °C samples. ^ii^: *** for the Control samples; * for the HPP samples. ^iii^: *** only for Control samples. ^iv^: only for 20 °C samples. ^v^: only for the HPP samples.

**Table 3:** Instrumental color and oxidative parameters (mean ± standard deviation) of sliced Iberian chorizo HPP-treated (HPP) or not (Control), not stored (0) or stored at 4 or 20 °C for 90 or 180 days, and significance from a one-way (storage time) ANOVA, a one-way (T, HPP) ANOVA on day 0 data, and a two-way (T, HPP; ST, storage temperature) ANOVA with interaction. For variables with significant interaction in Table 5, a Simple Main Effects test was performed. ^1^.

	Storage (Days)	4 °C		20 °C		*p*-Value		
		Control	HPP	Control	HPP	T	ST	TXST
CIE *L* *	0	46.7 ± 2.1	47.3 ± 2.1	46.7 ± 2.1	47.3 ± 2.1	ns	-	-
	90	46.0 ± 1.0	46.6 ± 3.0	47.3 ± 0.6	47.3 ± 1.6	ns	ns	ns
	180	46.6 ± 0.6	46.9 ± 0.4	47.0 ± 1.3	46.1 ± 1.4	ns	ns	ns
*p*-storage time		ns	ns	ns	ns			
CIE *a* *	0	16.0 ± 1.4	16.5 ± 1.2	16.0 ± 1.4	16.5 ± 1.2	ns	-	-
	90	15.9 ± 1.8	15.2 ± 1.9	15.7 ± 0.9	14.7 ± 2.1	ns	ns	ns
	180	15.6 ± 1.1	15.5 ± 1.7	14.5 ± 1.0	14.0 ± 2.1	ns	ns	ns
*p*-storage time		ns	ns	ns	ns			
CIE *b* *	0	12.3 ± 2.0	13.3 ± 1.7	12.3 ± 2.0	13.3 ± 1.7	ns	-	-
	90	11.7 ± 2.0	12.4 ± 3.3	11.5 ± 0.9	10.7 ± 2.6	ns	ns	ns
	180	10.9 ± 1.1	11.1 ± 1.3	11.1 ± 1.4	9.9 ± 2.2	ns	ns	ns
*p*-storage time		ns	ns	ns	ns			
**Chroma**	0	20.2 ± 2.3	21.3 ± 1.7	20.2 ± 2.3	21.3 ± 1.7	ns	-	-
	90	19.7 ± 2.7	19.6 ± 3.5	19.5 ± 1.3	18.2 ± 3.3	ns	ns	ns
	180	19.0 ± 1.5	19.1 ± 2.2	18.3 ± 1.7	17.2 ± 3.0	ns	ns	ns
*p*-storage time		ns	ns	ns	ns			
**Hue ^x^**	0	37.3 ± 2.8	38.8 ± 2.7	37.3 ± 2.8	38.8 ± 2.7	ns	-	-
	90	36.3 ± 1.8	38.5 ± 4.1	36.3 ± 0.9	35.8 ± 2.6	ns	ns	ns
	180	34.8 ± 1.1	35.6 ± 0.9	37.4 ± 1.8	35.0 ± 2.3	ns	ns	ns
*p*-storage time		ns	ns	ns	ns			
**MDA (mg Kg^−1^) ^x^**	0	0.4 b ± 0.1	0.3 b ± 0.1	0.4 b ± 0.1	0.3 b ± 0.1	ns	-	-
	90	0.4 b ± 0.1	0.5 a ± 0.1	0.5 ab ± 0.1	0.6 a ± 0.1	*^i^	ns	ns
	180	0.6 a ± 0.1	0.6 a ± 0.1	0.6 a ± 0.1	0.7 a ± 0.1	ns	ns	ns
*p*-storage time		*	**	*	**			
**Carbonyls (nmol mg protein^−1^) ^x^**	0	3.1 b ± 0.5	3.6 b ± 0.5	3.1 b ± 0.5	3.6 b ± 0.5	ns	-	-
	90	3.5 b ± 0.4	4.2 b ± 0.7	3.1 b ± 0.4	4.1 b ± 0.9	ns	ns	ns
	180	7.9 a ± 0.6	9.06 a ± 2.4	6.3 a ± 1.0	7.0 a ± 1.9	ns	*^ii^	ns
*p*-storage time		***	***	***	**			

^1^ ns: *p* ≥ 05; *: *p* < 0.05; **: *p* < 0.01; ***: *p* < 0.001. a, b: different letters in the same column indicate significant differences over storage in the Tukey test. ^x^: variable with significant interaction in Table 5, which underwent a Simple Main Effects test. ^i^: only in 4 °C samples. ^ii^: only in Control samples.

**Table 4:** Sensory characteristics (means ± standard deviation) of sliced Iberian chorizo HPP-treated (HPP) or not (Control), not stored (0) or stored at 4 or 20 °C for 90 or 180 days, and significance from a one-way (storage time) ANOVA, a one-way (T, HPP) ANOVA on day 0 data, and from a two-way (T, HPP; ST, storage temperature) ANOVA with interaction. For variables with significant interaction in Table 5, a Simple Main Effects test was performed.

	Storage (Days)	4 °C		20 °C		*p*-Value		
		Control	HPP	Control	HPP	T	ST	TXST
**Lean color ^x^**	0	6.9 ± 0.6	7.1 ± 0.6	6.9 ab ± 0.6	7.1 ± 0.6	ns	-	-
	90	6.8 ± 0.2	6.7 ± 0.5	6.6 b ± 0.5	6.9 ± 0.4	ns	ns	ns
	180	6.5 ^2^ ± 0.6	6.7 ^2^ ± 0.4	7.6 a ^1^ ± 0.3	7.6 ^1^ ± 0.5	ns	***	ns
*p*-storage time		ns	ns	*	ns			
**Odor intensity ^x^**	0	7.6 ± 0.4	7.3 ± 0.4	7.6 a ± 0.4	7.3 ± 0.4	ns	-	-
	90	7.2 ± 0.5	7.0 ± 0.1	6.9 b ± 0.2	7.2 ± 0.2	ns	ns	ns
	180	7.2 ± 0.1	7.0 ± 0.3	7.0 b ± 0.2	7.2 ± 0.5	ns	ns	ns
*p*-storage time		ns	ns	**	ns			
**Off-odor**	0	0.1 ± 0.1	0.0 ± 0.1	0.1 ± 0.1	0.0 b ± 0.1	ns	-	-
	90	0.1 ± 0.1	0.2 ± 0.3	0.1 ± 0.1	0.1 b ± 0.1	ns	ns	ns
	180	0.2 ± 0.2	0.2 ± 0.1	0.3 ± 0.2	0.4 a ± 0.4	ns	ns	ns
*p*-storage time		ns	ns	ns	*			
**Hardness ^x^**	0	4.5 ± 0.7	4.9 ± 0.6	4.5 b ± 0.7	4.9 ± 0.6	ns	-	-
	90	4.3 ± 0.9	4.9 ± 0.8	4.3 b ± 0.5	4.9 ± 0.3	ns	ns	ns
	180	4.5 ^3^ ± 0.3	4.9 ^2,3^ ± 0.2	5.5 a^1,2^ ± 0.4	5.6 ^1^ ± 0.6	*^,i^	**	ns
*p*-storage time		ns	ns	*	ns			
**Juiciness ^x^**	0	5.0 ± 0.6	4.9 ± 0.4	5.0 ± 0.6	4.9 ± 0.4	ns	-	-
	90	5.4 ± 0.5	5.0 ± 0.4	5.2 ± 0.5	5.0 ± 0.2	ns	ns	ns
	180	5.4 ^1^ ± 0.3	5.2 ^1^ ± 0.2	4.6 ^1,2^ ± 0.6	4.3 ^2^ ± 0.7	ns	**	ns
*p*-storage time		ns	ns	ns	ns			
**Saltiness (taste)**	0	5.0 ± 0.4	5.0 ab ± 0.1	5.0 ± 0.4	5.0 ± 0.1	ns	-	-
	90	5.2 ± 0.2	5.2 a ± 0.1	5.2 ± 0.2	5.3 ± 0.1	ns	ns	ns
	180	5.0 ± 0.3	4.6 b ± 0.5	4.7 ± 0.3	4.9 ± 0.4	ns	ns	ns
*p*-storage time		ns	*	ns	ns			
**Sourness (taste)**	0	5.0 a ± 0.1	5.0 a ± 0.3	5.0 a ± 0.1	5.0 a ± 0.3	ns	-	-
	90	4.6 a ± 0.4	4.5 a ± 0.4	4.5 b ± 0.2	4.5 b ± 0.2	ns	ns	ns
	180	3.6 b ± 0.4	3.6 b ± 0.4	3.5 c ± 0.4	3.6 c ± 0.2	ns	ns	ns
*p*-storage time		***	**	***	***			
**Sweetness (taste)**	0	4.7 a ± 0.2	4.7 a ± 0.3	4.7 a ± 0.2	4.7 a ± 0.3	ns	-	-
	90	4.5 a ± 0.3	4.5 a ± 0.4	4.5 a ± 0.4	4.5 a ± 0.4	ns	ns	ns
	180	2.9 b ± 0.6	2.9 b ± 0.4	2.9 b ± 0.2	2.9 b ± 0.5	ns	ns	ns
*p*-storage time		***	***	***	***			
**Spiciness**	0	4.3 ± 0.7	4.3 a ± 0.4	4.3 a ± 0.7	4.3 a ± 0.4	ns	-	-
	90	3.9 ± 0.9	4.2 a ± 0.7	3.8 ab ± 0.6	3.9 ab ± 0.6	ns	ns	ns
	180	3.5 ± 0.5	3.1 b ± 0.6	3.1 b ± 0.7	3.2 b ± 0.7	ns	ns	ns
*p*-storage time		ns	*	*	*			
**Flavor intensity ^x^**	0	7.2 ± 0.2	7.3 ± 0.2	7.2 a ± 0.2	7.3 ± 0.2	ns	-	-
	90	7.1 ± 0.3	7.0 ± 0.1	7.1 ab ± 0.2	7.1 ± 0.3	ns	ns	ns
	180	7.1 ± 0.2	7.0 ± 0.3	6.8 b ± 0.2	7.1 ± 0.2	ns	*^,ii^	ns
*p*-storage time		ns	ns	*	ns			
**Cured flavor**	0	4.2 b ± 0.4	4.4 ab ± 0.5	4.2 b ± 0.4	4.4 b ± 0.5	ns	-	-
	90	3.8 b ± 0.5	3.9 b ± 0.4	3.9 b ± 0.4	4.0 b ± 0.4	ns	ns	ns
	180	4.9 a ± 0.4	4.9 a ± 0.4	5.1 a ± 0.4	5.1 a ± 0.5	ns	ns	ns
*p*-storage time		**	*	**	**			
**Rancid flavor**	0	0.2 ± 0.1	0.2 ± 0.1	0.2 ± 0.1	0.2 ± 0.1	ns	-	-
	90	0.6 ± 0.3	0.7 ± 0.5	0.7 ± 0.5	0.8 ± 0.8	ns	ns	ns
	180	0.3 ± 0.3	0.4 ± 0.2	0.7 ± 0.4	0.6 ± 0.4	ns	ns	ns
*p*-storage time		ns	ns	ns	ns			

^1^ ns: *p* ≥ 0.05; *: *p* < 0.05; **: *p* < 0.01; ***: *p* < 0.001. a,b,c: different letters in the same column indicate significant differences over storage in the Tukey test. ^1,2,3^: different superscript numbers in the same row indicate significant differences between groups in the Tukey test. ^x^: variable with significant interaction in Table 5, which underwent a Simple Main Effects test. ^i^: only in 4 °C samples. ^ii^: only in Control samples.

**Table 5:** Significance from a three-way (HPP: T; storage temperature: ST; and storage time) ANOVA with interaction performed on the data from the samples stored for 90 and 180 days.

	T	ST	Time	TXST	ST X Time	T X Time	TXST X Time
Mesophilic aerobic bacteria	<0.001	<0.001	<0.001	<0.001	<0.001	<0.001	<0.001
Lactic acid bacteria	<0.001	<0.001	<0.001	0.060	0.001	0.620	0.014
Yeasts and molds	0.650	0.009	<0.001	0.032	0.022	0.940	0.013
Coliforms	0.051	<0.001	<0.001	0.051	<0.001	0.051	0.051
CIE *L* *	0.946	0.356	0.738	0.304	0.197	0.540	0.734
CIE *a* *	0.285	0.133	0.404	0.716	0.403	0.625	0.958
CIE *b* *	0.648	0.296	0.198	0.267	0.713	0.751	0.998
Chroma	0.442	0.190	0.282	0.461	0.799	0.931	0.989
Hue	0.956	0.791	0.145	0.043	0.100	0.232	0.917
MDA	0.026	0.123	0.001	0.807	0.871	0.182	0.654
Carbonyls	0.034	0.013	<0.001	0.884	0.049	0.866	0.636
Lean color	0.431	<0.001	0.020	0.548	0.001	0.918	0.315
Odor intensity	0.890	0.510	0.894	0.010	0.718	0.819	0.927
Off-odor	0.453	0.431	0.011	0.732	0.118	0.810	0.248
Hardness	0.019	0.015	0.005	0.694	0.027	0.356	0.649
Juiciness	0.045	0.005	0.048	0.845	0.016	0.782	0.516
Saltiness (taste)	0.694	0.968	<0.001	0.114	0.875	0.395	0.344
Sourness (taste)	0.818	0.498	<0.001	0.579	0.881	0.705	0.989
Sweetness (taste)	0.686	0.986	<0.001	0.948	0.908	0.961	0.879
Spiciness	0.992	0.440	0.001	0.590	0.960	0.483	0.405
Flavor intensity	0.782	0.559	0.197	0.048	0.361	0.604	0.575
Cured flavor	0.611	0.386	<0.001	0.887	0.774	0.753	0.793
Rancid flavor	0.899	0.189	0.168	0.705	0.631	0.872	0.709

## Data Availability

The authors confirm that the data supporting the findings of this study are available within the article and the raw data support the findings are available from the corresponding author, upon reasonable request.
